# Genomic Characterization of Invasive Meningococcal Serogroup B Isolates and Estimation of 4CMenB Vaccine Coverage in Finland

**DOI:** 10.1128/mSphere.00376-20

**Published:** 2020-09-16

**Authors:** Margherita Bodini, Alessandro Brozzi, Maria Giuliani, Hanna Nohynek, Anni Vainio, Markku Kuusi, Rosita De Paola, Mariagrazia Pizza, Duccio Medini, Maija Toropainen, Laura Serino, Alessandro Muzzi

**Affiliations:** a GSK, Siena, Italy; b Finnish Institute for Health and Welfare (THL), Helsinki, Finland; University of Maryland School of Medicine

**Keywords:** 4CMenB, MATS, antigen typing system, gMATS, genomics, meningococcal disease, vaccine coverage

## Abstract

Invasive meningococcal disease (IMD) caused by Neisseria meningitidis is a significant cause of morbidity and mortality worldwide. In Finland, the incidence rate of IMD is low, with meningococcal serogroup B (MenB) accounting for around one-third of IMD cases annually. The aim of this study was to investigate the genetic variability of invasive MenB isolates collected in Finland between 2010 and 2017 (*n* = 81), including the genes encoding the 4-component MenB vaccine (4CMenB; Bexsero; GSK) antigens and their promoters, and to evaluate the 4CMenB potential coverage. Whole-genome sequencing was performed. The meningococcal antigen typing system (MATS) was used to characterize MenB isolates and predict the potential coverage of 4CMenB. MATS was complemented by genetic MATS (gMATS) through association of antigen genotyping and phenotypic MATS results. Multilocus sequence typing revealed predominance of the ST-41/44 clonal complex among which sequence type (ST)-303 was the most common and was predicted to be covered by 4CMenB. Of the 4 major vaccine antigens, the factor H-binding protein variant 1, neisserial heparin binding antigen peptide 2, and the PorA P1.4 antigen were predominant, whereas *Neisseria* adhesin A was present in only 4% of the 81 isolates. MATS and gMATS 4CMenB strain coverage predictions were 78% and 86%, respectively, in a subpanel of 60 isolates collected during 2010 to 2014, with a gMATS prediction of 84% for all 81 isolates. The results suggest that 4CMenB could reduce the burden of IMD in Finland and that gMATS could be applied to monitor vaccine strain coverage and predict vaccine effectiveness.

**IMPORTANCE** 4CMenB is a 4-component vaccine used against invasive meningococcal disease (IMD) caused by Neisseria meningitidis serogroup B (MenB). We investigated the genetic variability of MenB in Finland and evaluated 4CMenB strain coverage by 2 different methods: MATS (meningococcal antigen typing system) and gMATS (genetic MATS). In a set of MenB isolates, 78% (MATS) and 86% (gMATS) were predicted as covered by 4CMenB, suggesting that use of 4CMenB would help reduce IMD incidence in Finland. MATS has been used in 13 countries worldwide, generating information on phenotypic characteristics that could infer protection by 4CMenB. Based on these data and genetic information, gMATS coverage predictions can be made. gMATS predicts coverage consistent with MATS for about 94% of tested strains. Unlike MATS, gMATS does not require live isolates, thus allowing the analysis also of noncultivable strains, making the coverage predictions more accurate. Therefore, gMATS can replace MATS to assess 4CMenB coverage, including in regions with no prior MATS data.

## Abstract (precis)

4CMenB is a 4-component vaccine used against invasive meningococcal disease (IMD) caused by Neisseria meningitidis serogroup B (MenB). We investigated the genetic variability of MenB in Finland and evaluated 4CMenB strain coverage by 2 different methods: MATS (meningococcal antigen typing system) and gMATS (genetic MATS). In a set of MenB isolates, 78% (MATS) and 86% (gMATS) were predicted as covered by 4CMenB, suggesting that use of 4CMenB would help reduce IMD incidence in Finland. MATS has been used in 13 countries worldwide, generating information on phenotypic characteristics that could infer protection by 4CMenB. Based on these data and genetic information, gMATS coverage predictions can be made. gMATS predicts coverage consistent with MATS for about 94% of tested strains. Unlike MATS, gMATS does not require live isolates, thus allowing the analysis also of noncultivable strains, making the coverage predictions more accurate. Therefore, gMATS can replace MATS to assess 4CMenB coverage, including in regions with no prior MATS data.

## INTRODUCTION

Neisseria meningitidis (meningococcus) is a Gram-negative bacterium that causes invasive meningococcal diseases (IMD) (1) such as meningitis and septicemia and may rapidly progress from early symptoms to death within 24 to 48 h. Twelve meningococcal serogroups (Men) have been identified, of which serogroup A (MenA), MenB, MenC, MenW, MenX, and MenY are most prevalent among disease cases worldwide (2). However, IMD epidemiology and serogroup distribution vary by time and geographic location (1).

In Finland, the incidence rate of IMD has significantly decreased during the past 2 decades (3) and was as low as 0.29 cases per 100,000 inhabitants of all ages, but reaching 1.88 cases per 100,000 population in infants in 2017 (4). Due to the low incidence of IMD, meningococcal vaccines have not been introduced into the national vaccination program in Finland, except for persons at higher risk of invasive disease due to certain medical conditions including complement deficiencies or anatomic or functional asplenia. Military conscripts are also eligible for MenACWY vaccination upon entry into service (3). The majority of IMD cases (51%) reported by European countries to the European Centre for Disease Prevention and Control in 2017 were caused by MenB. In Finland, MenB caused at least one-third of IMD cases annually between 2010 and 2016 and was the second most prevalent serogroup after MenY in 2017, accounting for approximately 19% of IMD cases (4).

A 4-component MenB vaccine (4CMenB; Bexsero; GSK) has been developed to prevent IMD caused by MenB and is licensed from the age of 2 months onward in the European Union and several countries worldwide and for individuals 10 to 25 years of age in the United States. It consists of 4 major antigenic components, the factor H-binding protein (fHbp), *Neisseria* adhesin A (NadA), neisserial heparin binding antigen (NHBA), and outer membrane vesicles from the New Zealand outbreak strain NZ98/254 (NZ OMV) expressing PorA P1.4, as the major antigen (5). The vaccine induces bactericidal antibody response against a wide range of MenB strains and has been proven effective in preventing MenB disease in infants and adolescents (6–10).

Given the high variability of *Neisseria* surface proteins and their level of expression, predicting potential effectiveness of MenB vaccines has been challenging. A titer ≥4 in serum bactericidal activity assay using human complement (hSBA) (11) is the accepted correlate of protection that is used in the clinical evaluation and licensure of MenB vaccines (12). Currently, the immunogenicity of MenB vaccines is evaluated against a limited number of strains. In prelicensure studies, several indicator strains were used, chosen to investigate serum bactericidal activity against each of the major antigens in 4CMenB (13). However, the susceptibility of a strain to vaccine-induced antibodies depends on several factors, of which the most important are the similarity of antigens present in the meningococcus and the vaccine and the levels of expression of the respective antigens in the tested strain. Due to genetic variability and expression of neisserial antigens across strains, a comprehensive evaluation of a MenB vaccine coverage would require the testing of a large number of isolates using hSBA. This would be laborious and would require large volumes of serum and human complement; therefore, alternative methods to evaluate strain coverage were pursued.

A meningococcal antigen typing system (MATS) was developed and standardized across public health laboratories worldwide (14, 15) to predict the potential coverage of 4CMenB (14) in different countries. The MATS combines a sandwich enzyme-linked immunosorbent assay (ELISA) for detection of qualitative and quantitative differences in fHbp, NadA, and NHBA expression together with PorA genotyping (14). The MATS results correlate with killing of strains in hSBA (14). The MATS serves as a conservative predictor of strain coverage by 4CMenB in infants and adolescents (16) and has been employed to predict 4CMenB strain coverage in many countries (17–21). It has recently been shown that MATS can be complemented by genetic MATS (gMATS) through association of antigen genotyping and phenotypic MATS results (22). The gMATS is emerging as a cost-effective tool which has the added benefit of not requiring cultivable isolates, thus allowing prediction across MenB-caused IMD cases identified by PCR only.

Recent studies also demonstrated that the sequence analysis of intergenic regions (IRs) may be informative for assessing fHbp protein expression and strain coverage of protein-based MenB vaccines (23, 24).

The aim of this study was to investigate the genetic variability of invasive MenB isolates collected in Finland between 2010 and 2017, including the analysis of the 4CMenB vaccine antigens and their promoters, and to evaluate 4CMenB coverage in the same panel of isolates.

## RESULTS

### Isolate collection.

Notification of laboratory-confirmed IMD to the National Infectious Disease Register (NIDR) has been statutory in Finland since 1995, and all IMD case isolates are requested to be sent to the reference laboratory at the Finnish Institute for Health and Welfare (THL) for species verification and strain characterization. Data were collected by epidemiological years (from July through June of the subsequent year). From 2010/2011 to 2016/2017, a total of 177 laboratory-confirmed IMD cases were reported to the NIDR. Of these, 94% (167 cases) were confirmed by culture and 6% (10 cases) by detection of N. meningitidis nucleic acid in cerebrospinal fluid. Forty-six percent (81) of all cases were caused by MenB; all were confirmed by blood or cerebrospinal fluid culture. For the present study, all MenB IMD isolates were analyzed.

Whole-genome sequence data were used to characterize the isolates, including conventional 7-locus multilocus sequence typing (MLST), core genome MLST (cgMLST), and sequencing of 4CMenB antigens (as defined by the *Neisseria* PubMLST database [https://www.pubmlst.org]), and fHbp and NHBA IRs.

### MLST.

**FIG 1:**
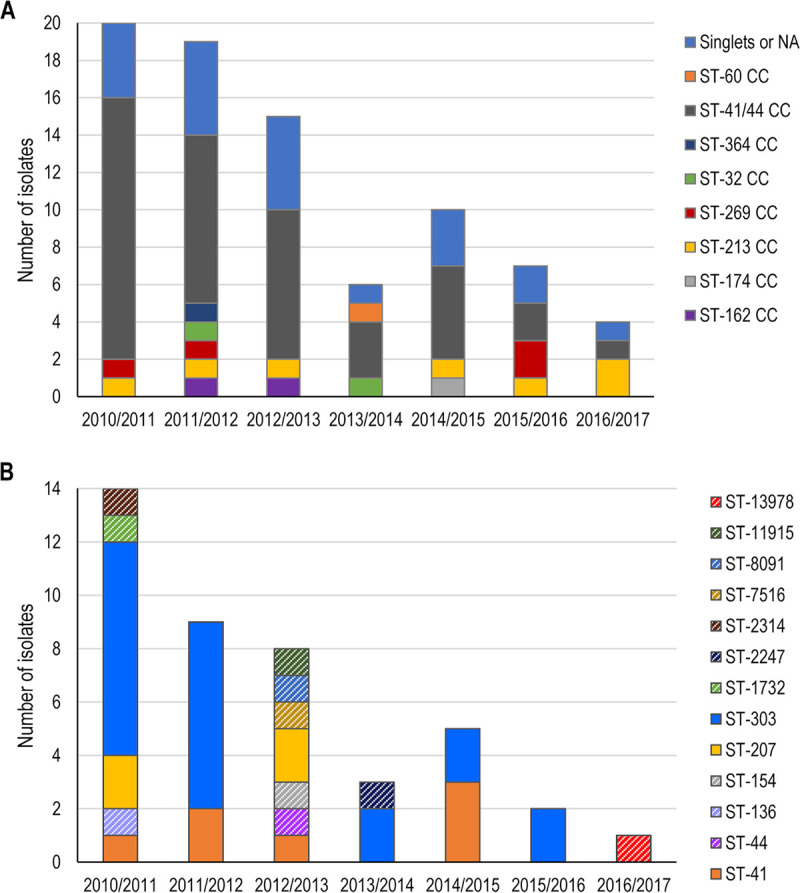
Clonal complex distribution for MenB strains in Finland (A) and sequence type distribution within the ST-41/44 clonal complex (B), by epidemiological years. NA, not available; ST, sequence type; MenB, meningococcal serogroup B; CC, clonal complex. Note: in panel B, STs appearing only once are represented with a diagonal stripe pattern to emphasize the recurrent STs (ST-41, ST-207, and ST-303).

The sequence type (ST)-41/44 clonal complex (CC) was the most predominant in Finland during the study period, although with a decreasing trend over time, overall accounting for 52% (42/81) of the isolates. The ST-213 CC was also identified during 3 years of the covered period, although only 1 to 2 isolates/year belonged to this CC. Other isolates were singlets, or the CC assignment was not possible (Fig. 1A). Thirteen different STs were associated with the ST-41/44 CC, among which ST-303 (21/42, 50%) with the antigen profile B:P1.7-2,4:F1-5 was the most common (Fig. 1B).

To evaluate the frequency and distribution of ST-303, we analyzed the *Neisseria* PubMLST database and identified 49 ST-303 isolates from different countries of which 10 were defined as carrier and 39 as invasive. Five of the 9 Finnish IMD isolates present in the public database, collected during 2015 to 2016, were also included in this study, while the remaining 4 were isolated in 2000, 2002, and 2018 and thus were outside the period analyzed in this study. In the PubMLST database, most isolates (10/19) collected from 1971 to 2000 were carrier strains, while between 2001 and 2018, all were invasive (see Fig. S1 in the supplemental material).

10.1128/mSphere.00376-20.1 FIG S1 ST-303 isolates in the *Neisseria* PubMLST database (worldwide). ST, sequence type; IMD, invasive meningococcal disease. Note: bar graph indicates the number of public carrier (turquoise column) or invasive (red column) isolates per year. Download FIG S1, DOCX file, 0.02 MB. Copyright © 2020 Bodini et al. 2020 Bodini et al. This content is distributed under the terms of the Creative Commons Attribution 4.0 International license.

### Phylogenetics.

**FIG 2:**
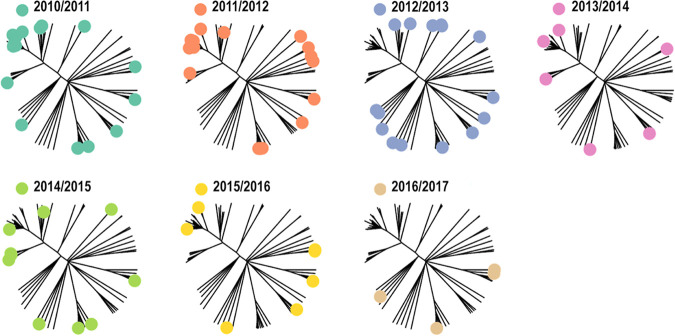
Phylogenetic tree based on the core genome MLST scheme from PubMLST of 81 Finnish MenB strains. MLST, multilocus sequence typing; MenB, meningococcal serogroup B. Note: in each panel, strains appearing in each epidemiological year are displayed as colored dots. A detailed description of the algorithms used for tree construction can be found in Materials and Methods.

Based on cgMLST analysis, we reconstructed the phylogeny of the MenB isolates, refining the information that is usually analyzed at ST level. Figure 2 shows the phylogenetic tree for each epidemiological year, illustrative of the genetic diversity of isolates causing IMD in Finland. Closely related isolates sharing similar core genomes were identified in multiple epidemiological years, showing the persistence of several clones over time.

**FIG 3:**
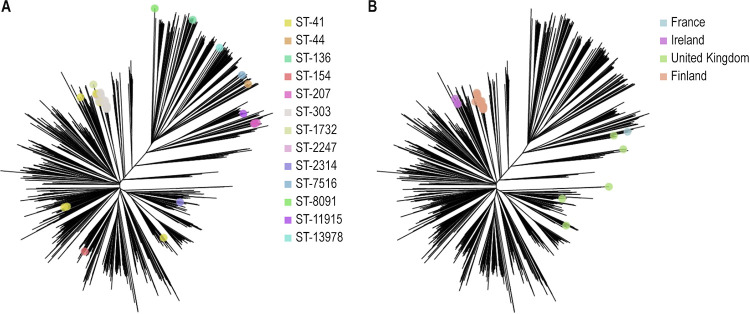
Phylogenetic tree based on the core genome MLST scheme from PubMLST including 42 Finnish ST-41/44 CC strains (A) and 1,109 public European ST-41/44 CC MenB strains (B). MLST, multilocus sequence typing; ST, sequence type; CC, clonal complex; MenB, meningococcal serogroup B; UK, United Kingdom. Note: dots represent Finnish strains, colored by their ST, in panel A and ST-303 strains, colored by their country of origin, in panel B. A detailed description of the algorithms used for tree construction can be found in Materials and Methods.

We compared the genomics of the ST-41/44 CC in Finland with the rest of Europe through the phylogenetic analysis of the cgMLST of 1,109 public strains downloaded from the PubMLST. Finnish strains were distributed all around the phylogram; among them, ST-303 isolates represented a small cluster, separated from the rest of the graph (Fig. 3A). Finnish ST-303 isolates were distant from ST-303 strains identified in other European countries (Ireland, France, and United Kingdom, Fig. 3B).

### Antigen sequence characterization.

Antigen sequence characterization revealed that fHbp variant 1 (subfamily B) was present in 74% (60/81) of isolates, with subvariant 14 as the most prevalent (33%, 27/81), followed by subvariants 4 and 13, each detected in 14% (11/81) of isolates. The NHBA peptide 2, included in 4CMenB, was detected in 35% (28/81) of isolates and was the most common NHBA variant among the isolates, while peptide 20 was present in 15% (12/81) of isolates. NadA was present in 4% (3/81) of isolates, always presenting peptide 1 (Table 1). Genetic typing of the PorA variable region revealed that PorA subtype P1.4 matching the vaccine variant was present in 30% (24/81) of isolates. Sequence characterization further demonstrated that fHbp and NHBA peptide sequences showed a strong association with ST and CC and between each other (*P* values <1 × 10^−6^) and that NadA showed significant association with ST-32 (very few positives).

**TABLE 1:** Summary of 4CMenB antigen sequence characterization for MenB strains in Finland^*a*^

CC	ST	fHbp variant	fHbp peptide	NHBA peptide	NadA peptide	PorA_VR2	No. of isolates
ST-162 complex	10538	3	31	20	X	15	2
ST-174 complex	2272	1	4	9	X	9	1
ST-213 complex	213	3	45	18	X	14	1
ST-213 complex	213	3	45	1341	X	14	1
ST-213 complex	213	3	45	29	X	14	1
ST-213 complex	213	3	45	1057	X	14	1
ST-213 complex	213	3	882	18	X	14	1
ST-213 complex	213	3	187	637	X	14	1
ST-213 complex	13348	3	45	1342	X	14	1
ST-269 complex	269	1	15	21	X	2-2	1
ST-269 complex	2693	1	15	21	X	13-1	1
ST-269 complex	479	1	15	21	X	13-1	1
ST-269 complex	10943	2	19	1058	X	13	1
ST-32 complex/ET-5 complex	32	1	883	3	1	16-6	1
ST-32 complex/ET-5 complex	32	1	1	3	1	16	1
ST-364 complex	7523	1	2	635	X	13-1	1
ST-41/44 complex	41	1	4	2	X	4	1
ST-41/44 complex	41	1	14	2	X	10-7	1
ST-41/44 complex	41	1	14	894	X	10-7	1
ST-41/44 complex	303	1	14	2	X	4	3
ST-41/44 complex	303	1	14	2	X	16-3	1
ST-41/44 complex	13978	1	13	1344	1	13-1	1
ST-41/44 complex/lineage 3	41	1	4	2	X	9	1
ST-41/44 complex/lineage 3	41	1	4	2	X	4	1
ST-41/44 complex/lineage 3	41	1	14	2	X	10-7	1
ST-41/44 complex/lineage 3	41	1	14	129	X	1	1
ST-41/44 complex/lineage 3	303	1	14	2	X	4	16
ST-41/44 complex/lineage 3	303	1	14	1056	X	4	1
ST-41/44 complex/lineage 3	207	2	19	1055	X	14-6	1
ST-41/44 complex/lineage 3	207	2	19	43	X	14-6	3
ST-41/44 complex/lineage 3	7516	1	525	266	X	13-4	1
ST-41/44 complex/lineage 3	8091	NA (chimera)	207	892	X	28	1
ST-41/44 complex/lineage 3	2247	1	14	2	X	10-7	1
ST-41/44 complex/lineage 3	154	1	4	10	X	4	1
ST-41/44 complex/lineage 3	11915	2	19	2	X	15-1	1
ST-41/44 complex/lineage 3	44	1	8	2	X	26	1
ST-41/44 complex/lineage 3	136	2	24	10	X	16-3	1
ST-41/44 complex/lineage 3	2314	1	4	31	X	4	1
ST-41/44 complex/lineage 3	1732	1	110	894	X	25-11	1
ST-60 complex	4146	1	13	24	X	2	1
NA	NA	1	13	20	X	14	1
NA	NA	3	31	20	X	15	1
Singlet	1572	1	13	20	X	14	6
Singlet	1572	1	13	20	X	10-1	1
Singlet	1572	1	13	1343	X	14	1
Singlet	10184	1	14	21	X	14-6	1
Singlet	11515	1	260	20	X	14	1
Singlet	2271	1	135	46	X	10-2	1
Singlet	2793	NA	Contig border	893	X	34	1
Singlet	2793	1	4	893	X	34	2
Singlet	2793	1	4	893	X	2-2	1
Singlet	11916	1	4	664	X	1	1
Singlet	9390	2	24	6	X	1	1
Singlet	2265	1	4	893	X	14	1
Singlet	10942	2	106	21	X	3	1

a Abbreviations: 4CMenB, multicomponent meningococcal B vaccine; CC, clonal complex; ST, sequence type; fHbp, factor H-binding protein; NHBA, neisserial heparin binding antigen; NadA, *Neisseria* adhesin A; PorA, porin A; VR, variable region; NA, not available.

### Strain coverage prediction by MATS and gMATS.

**FIG 4:**
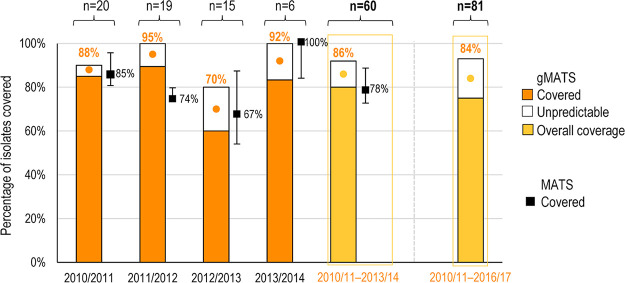
Strain coverage predicted by gMATS and MATS. (g)MATS, (genetic) meningococcal antigen typing system. Note: the percentages indicated above bar plots are the point estimates of gMATS coverage for every epidemiological year, calculated assuming that half of the unpredictable strains are covered. The values at the right of the dot-and-whisker plots indicate the percentage of strains predicted to be covered by 4CMenB in MATS; whiskers represent the corresponding 95% confidence intervals for MATS estimates.

When the 60 strains isolated between 2010/2011 and 2013/2014 were analyzed by MATS, the predicted strain coverage of 4CMenB was 78% (95% confidence interval [CI], 72% to 88%). Coverage prediction by MATS was not available for the 21 MenB isolates recovered between 2014/2015 and 2016/2017 (Fig. 4), while the genotyping was possible, allowing estimation of gMATS prediction. The 4CMenB strain coverage predicted by gMATS for the 60 isolates collected during the epidemiological years from 2010/2011 to 2013/2014 was 86% (lower limit to upper limit: 80% to 92%). For the epidemiological year 2011/2012, MATS and gMATS coverage predictions were 74% and 95%, respectively. Thus, the 5 out of 19 isolates predicted as not covered by MATS were predicted as covered (3 isolates) or unpredictable (2 isolates) by gMATS. gMATS predictions for these 5 strains relied only on NHBA. Isolates presenting peptides 20 and 21 were positive in gMATS, while isolates carrying peptides 1057 and 1058 were unpredictable. In the same epidemiological year, peptide 20 was present in 3 other strains that were predicted as covered for NHBA by MATS. Overall, after the inclusion of the 21 strains collected from 2014/2015 to 2016/2017, the gMATS predicted coverage was 84% (lower limit to upper limit: 75% to 93%), without a temporal trend within a 2-year nonoverlapping window (Fig. 4).

**FIG 5:**
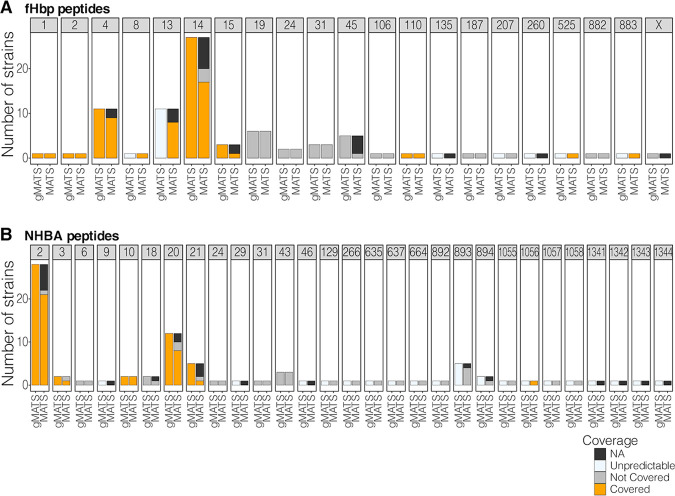
Strain coverage predicted by gMATS and MATS for fHbp (A) and NHBA (B) peptides. (g)MATS, (genetic) meningococcal antigen typing system; CB, contig border; fHbp, factor H-binding protein; NA, not available (for isolates collected in the epidemiological years from 2014/2015 to 2016/2017); NHBA, neisserial heparin binding antigen.

4CMenB coverage could be predicted with gMATS in 88% of the isolates, with an accuracy of 94% compared to MATS. gMATS and MATS coverage predictions by antigen peptides are shown in Fig. 5, coverage prediction by ST is shown in Fig. S2, and a comparison between MATS and gMATS predictions is detailed in Table S1 in the supplemental material. The sensitivity, specificity, and positive and negative predictive values of gMATS versus MATS were 100%, 62.5%, and 93.8% and 100%, respectively.

10.1128/mSphere.00376-20.2 FIG S2 gMATS and MATS coverage predictions for each sequence type. (g)MATS, (genetic) meningococcal antigen typing system; NA, not available (for isolates collected in the epidemiological years from 2014/2015 to 2016/2017). Download FIG S2, DOCX file, 0.09 MB. Copyright © 2020 Bodini et al. 2020 Bodini et al. This content is distributed under the terms of the Creative Commons Attribution 4.0 International license.

10.1128/mSphere.00376-20.6 TABLE S1 Comparison between MATS and gMATS predictions. (g)MATS, (genetic) meningococcal antigen typing system. Note: comparison of MATS and gMATS predictions was done for 60 isolates collected during epidemiological years 2010/2011 to 2013/2014. Download Table S1, DOCX file, 0.01 MB. Copyright © 2020 Bodini et al. 2020 Bodini et al. This content is distributed under the terms of the Creative Commons Attribution 4.0 International license.

All ST-303 isolates were predicted to be covered by 4CMenB in MATS and gMATS. Fifteen of the 17 isolates tested by MATS and 19 of the 21 isolates assessed with gMATS were predicted to be covered by the three antigens fHbp, NHBA, and PorA together, while the remaining were covered by 2 antigens simultaneously.

### IR analysis and MATS relative potency.

The coding sequences of both fHbp and NHBA were associated with their IRs. The same IR cooccurred with different antigen peptides, while the opposite was rarely found (Fig. S3 and S4).

10.1128/mSphere.00376-20.3 FIG S3 Correspondence between fHbp peptide sequences and intergenic regions fHbp, factor H-binding protein. Note: blue cells show the number of isolates carrying that combination of peptide and intergenic allele. Darker shades of blue indicate more frequent combinations of fHbp peptide with the intergenic region. Download FIG S3, DOCX file, 0.1 MB. Copyright © 2020 Bodini et al. 2020 Bodini et al. This content is distributed under the terms of the Creative Commons Attribution 4.0 International license.

10.1128/mSphere.00376-20.4 FIG S4 Correspondence between NHBA peptide sequences and intergenic regions. NHBA, neisserial heparin binding antigen. Note: blue cells show the number of isolates carrying that combination of peptide and intergenic allele. Darker shades of blue indicate more frequent combinations of NHBA peptide with the intergenic region. Download FIG S4, DOCX file, 0.08 MB. Copyright © 2020 Bodini et al. 2020 Bodini et al. This content is distributed under the terms of the Creative Commons Attribution 4.0 International license.

fHbp peptides cooccurred usually with the same *fHbp* IR, except for peptides 4, 14, 15, and 45, which were identified in isolates carrying different *fHbp* IRs (multiple colored cells on the same line in Fig. S3). For fHbp peptides 4 and 14, the majority of the isolates were covered (above the positive bactericidal threshold), but the effect of IRs on peptide expression could not be assessed, because of the limited number of isolates available per peptide-IR combination (Fig. S5).

10.1128/mSphere.00376-20.5 FIG S5 Relative potencies of isolates carrying the same fHbp peptide and diverse IRs. fHbp, factor H-binding protein; IR, intergenic region; PBT, positive bacterial threshold; MATS, meningococcal antigen typing system. Note: the red line corresponds to the MATS PBT for fHbp. The phylograms indicate intergenic regions found with fHbp peptide 14 (left panel) and peptide 4 (right panel). The MATS PBT used for fHbp is 0.012. Download FIG S5, DOCX file, 0.05 MB. Copyright © 2020 Bodini et al. 2020 Bodini et al. This content is distributed under the terms of the Creative Commons Attribution 4.0 International license.

Isolates expressing a specific NHBA peptide always carried the same NHBA IR sequence (Fig. S4); therefore, the impact of IR on protein expression could not be assessed.

## DISCUSSION

In the current study, we evaluated the genetic variability of invasive MenB isolates recovered in Finland in 2010 to 2017 and assessed the predicted coverage of 4CMenB. IMD caused by MenB was endemic in Finland and was characterized by the predominance of the ST-41/44 CC with ST-303 being the most common ST and being covered by 4CMenB.

In previous studies assessing the coverage of 4CMenB in other European countries, the ST-41/44 CC predominated also in England and Wales (31.6%), France (40.5%), Germany (40.1%), Norway (51.2%), and Italy (55.5%) (17). Similarly, in studies performed in the United States and Canada, the ST-41/44 CC was among the most frequently isolated CCs (20, 21). A study performed in Finland during 2004 to 2006 demonstrated that MenB was significantly associated with the ST-41/44 CC in both carrier and invasive isolates (25). Based on previous analysis (26) and the results of our study, isolates belonging to the ST-41/44 CC have circulated in Finland since the mid-1990s until 2018, with gradually decreasing frequency over time.

Among the ST-41/44 CC, ST-303, which is uncommon in other countries and identified in the past from both healthy carriage and invasive disease isolates (27), was the most abundant ST in this strain collection and was predicted to be covered by 4CMenB. From PubMLST data, a change in the proportions of carrier and invasive strains was apparent, switching from preferentially carrier (1971 to 2000) to preferentially invasive in more recent years (1988 to 2015). However, this interpretation is hindered by sampling biases and the fact that the database is not representative of the global genomic diversity of MenB strains, as most isolates are deposited from specific studies and not systematically collected from all regions of the world.

MATS estimates may vary distinctively with the period of time for which the prediction was obtained and the country. Globally, 4CMenB coverage predictions ranged from 66% in the UK (95% CI: 52% to 80%) during 2014 to 2015 to 91% (95% CI: 72% to 96%) in the United States (2000 to 2008) (21, 28). In European countries, MATS 4CMenB strain coverage prediction reached 88% (95% CI: 60 to 96) in Greece during 2008 to 2010 (29).

In the current study, MATS and gMATS coverage predictions were high and concordant (78% [95% CI: 72% to 88%] and 86% [lower limit to upper limit: 80% to 92%], respectively). The main source of discrepancy for the predictions came from the gMATS-unpredictable isolates, for which a gross coverage of 50% is assumed in gMATS, as previously described (22). However, for this set of isolates, a coverage of 29% was estimated in MATS in this study. The assumption of 50% coverage for unpredictable isolates might be inaccurate in small data sets and is under refinement for future gMATS implementation. Moreover, characteristics other than peptide sequence may have impact on MATS coverage, as shown by the discrepancy noted between NHBA coverage estimated by MATS and gMATS for 2011/2012. Nevertheless, the high concordance on predictable isolates (94%) between MATS and gMATS results confirms that gMATS can be a useful tool to assess strain coverage cost-effectively, with the additional benefit over MATS of being applicable also to PCR-positive, noncultivable cases, which in certain countries can constitute half of the reported cases (22). These results also suggest that gMATS could be an effective strategy to ensure ongoing monitoring of the impact of 4CMenB vaccination on meningococcal disease epidemiology, an important public health task associated with significant challenges (30).

The close genetic relatedness between the Finnish MenB isolates suggests descendance from a small set of bacteria, possibly spreading through the population and occasionally leading to invasive disease and perhaps also linked to the occurrence of the majority of IMD cases in the most densely populated areas of Southern Finland. Moreover, this could have been enhanced by the geographic location (distant from other European countries) and the high prevalence of the B:P1.7-2,4:F1-5 (ST-41/44 CC) clone during the study period.

As revealed through antigen sequence characterization, alleles of the antigens that may be recognized by 4CMenB-induced antibody response to fHbp, NHBA, and PorA were predominant. Complete and in-frame sequence for NadA was present in only 4% of the isolates.

Biagini et al. recently demonstrated an association between promoter sequence clades and different levels of fHbp expression (24). Another study of promoter-containing IR found specific fHbp peptide-IR expression cluster combinations in multiple IMD cases. It was therefore suggested that sequence-based analysis of IR sequences is informative for assessing protein expression and can potentially be used to assess strain coverage by protein-based vaccines (23). However, in our data set, we have found very few cases where the same promoter was associated with different IRs. Therefore, in our study, it was challenging to draw any conclusions on the impact of IRs on protein expression and, consequently, on predicted 4CMenB protection. The investigation of a possible role of the IR in the expression level of fHbp was limited by the fixed peptide-IR associations, which did not allow us to dissect the impact of antigen expression level, possibly determined by the IR, and the similarity to the vaccine antigen peptide on MATS relative potency.

IMD caused by MenB in Finland was characterized by the predominance of the ST-41/44 CC and more specifically, ST-303, an ST predicted to be covered by 4CMenB. fHbp variant 1 group, NHBA, and PorA matching the vaccine peptides were predominant, while NadA was present in a low proportion of the isolates. MATS and gMATS 4CMenB strain coverage predictions for Finland were high (78% and 86%, respectively) and concordant, suggesting that the use of 4CMenB could lead to a reduction in IMD cases and that gMATS could be used to predict the impact of 4CMenB vaccination on meningococcal epidemiology.

## MATERIALS AND METHODS

### Whole-genome sequencing.

The 60 isolates collected during epidemiological years 2010/2011 to 2013/2014 were sequenced with support from GSK. The library preparation was done with the Nextera DNA kit (Illumina), and sequencing was performed with an Illumina HiSeq 1500 Flow Cell v3 system to run 2 × 100 paired-end (PE) reads. Read cleaning was performed by PhiX removal with Bowtie2 and by read trimming with Trimmomatic. Assembly was performed with SPAdes.

The 21 isolates collected from 2014/2015 to 2016/2017 were sequenced at the THL. The library preparation was done with the Nextera DNA kit (Illumina), and sequencing was performed using the Illumina Miseq2 platform to run 2 × 150 PE reads. Assembly was performed with SPAdes.

### 4CMenB coverage prediction.

The potential coverage of 4CMenB was assessed by exploring antigen expression and cross-reactivity through MATS (only for epidemiological years 2010/2011 to 2013/2014), and antigen sequences through gMATS (epidemiological years 2010/2011 to 2016/2017), as previously described (14, 22).

### IR sequence analysis of *fHbp* and *nhba*.

The upstream IRs of the *fHbp* and *nhba* genes were considered for an analysis of their association with the variety of protein peptides encoded by the 2 genes. The IR locus was defined as previously described for fHbp (24) and as the entire tract of noncoding DNA between the gene preceding the gene of interest on the closed reference genome MC58 for NHBA. This was possible because both the regions upstream of *fHbp* and *nhba* are very well conserved in N. meningitidis. The presence of different peptide-IR combinations in *fHbp* and *nhba* was assessed. In cases where the same peptide was associated with multiple IRs, the respective MATS relative potencies were compared to assess the impact of IR on protein expression.

### Phylogenetic analysis.

Phylogenetic analyses were performed on the cgMLST data using Jaccard’s distance to compare the strain profiles and using the Neighbor Joining function from the ape R package to build the phylogram. ST-41/44 CC cgMLST profiles of strains isolated in Europe have been downloaded from the PubMLST database updated until March 2019.

### Trademark statement.

Bexsero is a trademark owned by or licensed to the GSK group of companies.

### Data availability.

GSK makes available the anonymized individual participant data and associated documents from interventional clinical studies which evaluate medicines, upon approval of proposals submitted to www.clinicalstudydatarequest.com. To access data for other types of GSK-sponsored research, for study documents without patient-level data and for clinical studies not listed, please submit an enquiry via the website.
