# Supplementary figures and images for: Genomic Characterization of Invasive Meningococcal Serogroup B Isolates and Estimation of 4CMenB Vaccine Coverage in Finland
Source: mSphere. 2020 Sep 16;5(5):e00376-20. doi: 10.1128/mSphere.00376-20 (PMC7494829; doi:10.1128/mSphere.00376-20)

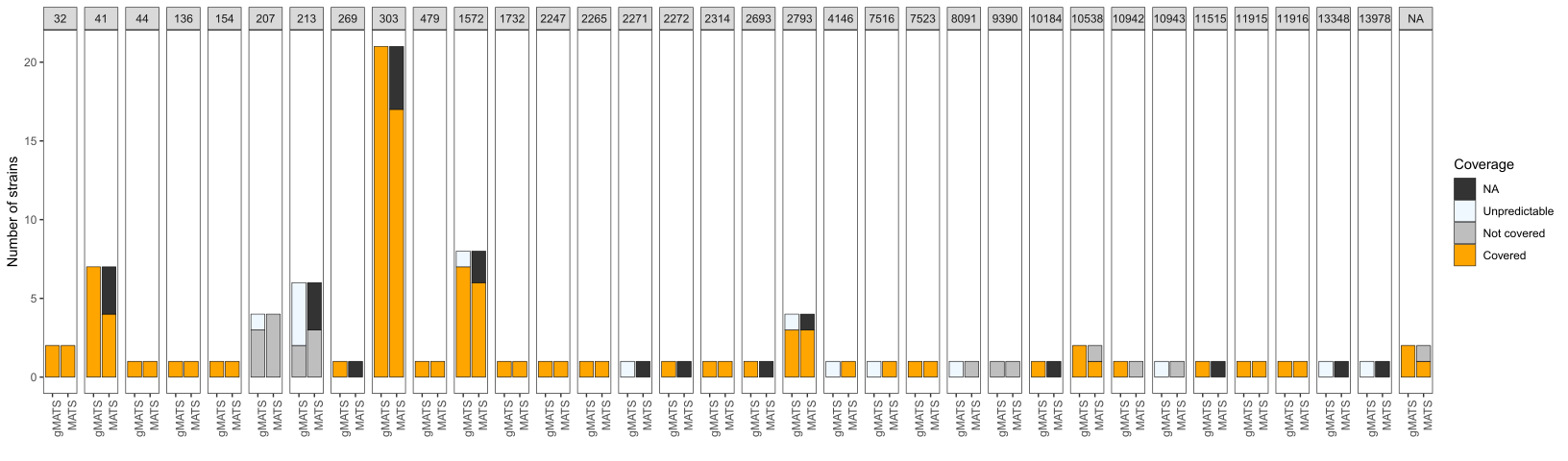

Supplement: FIG S2 [file mSphere.00376-20-sf002.docx]

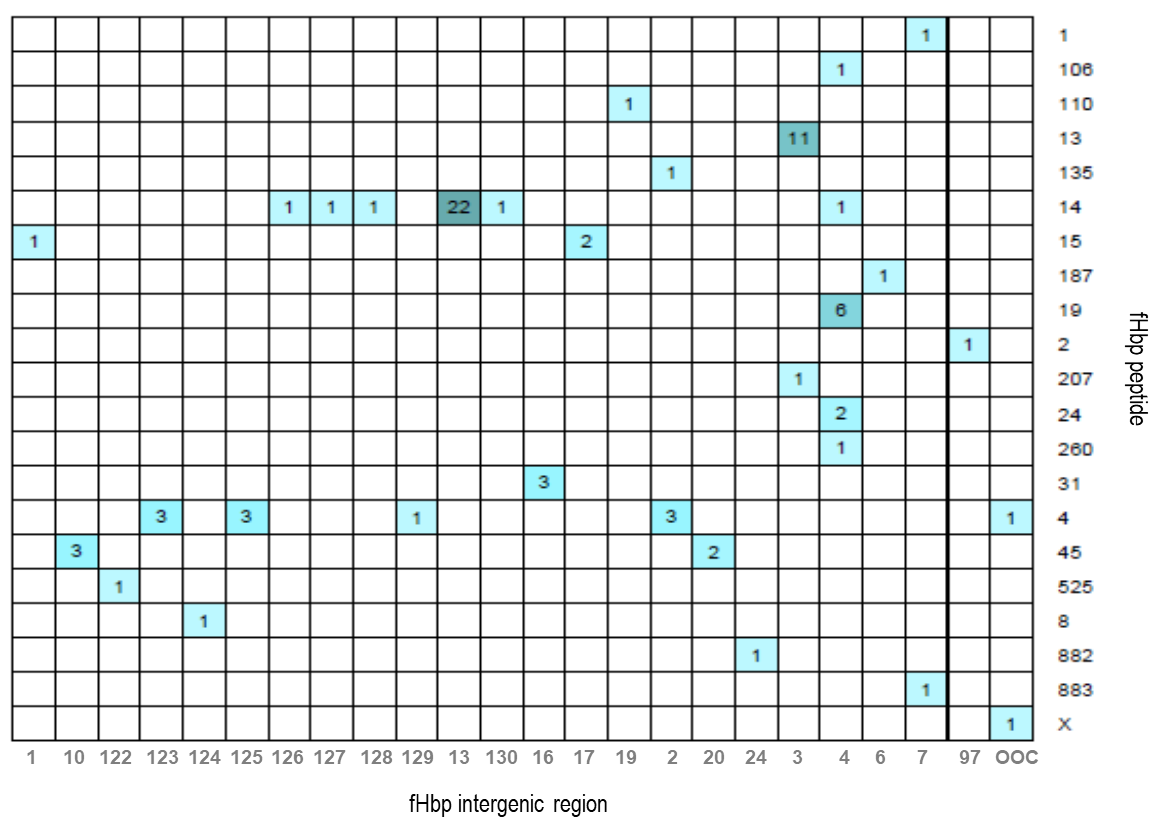

Supplement: FIG S3 [file mSphere.00376-20-sf003.docx]

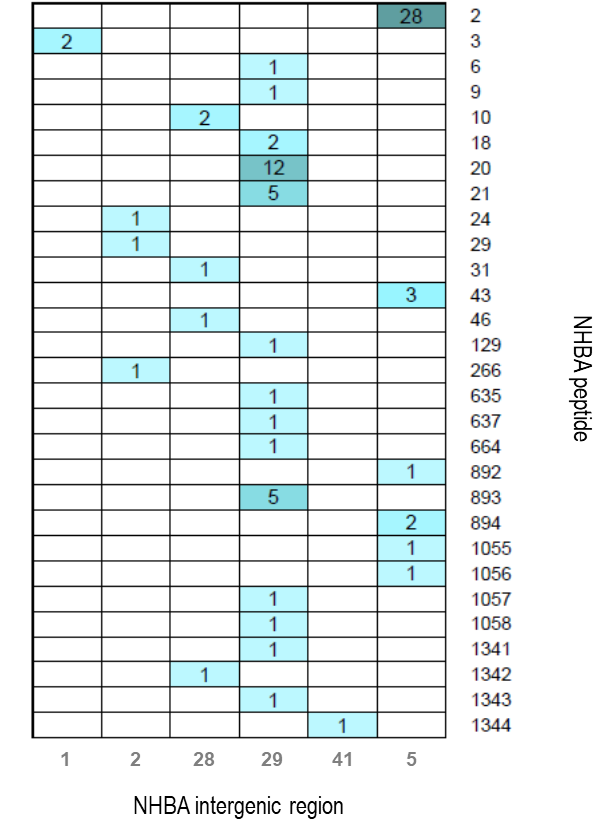

Supplement: FIG S4 [file mSphere.00376-20-sf004.docx]

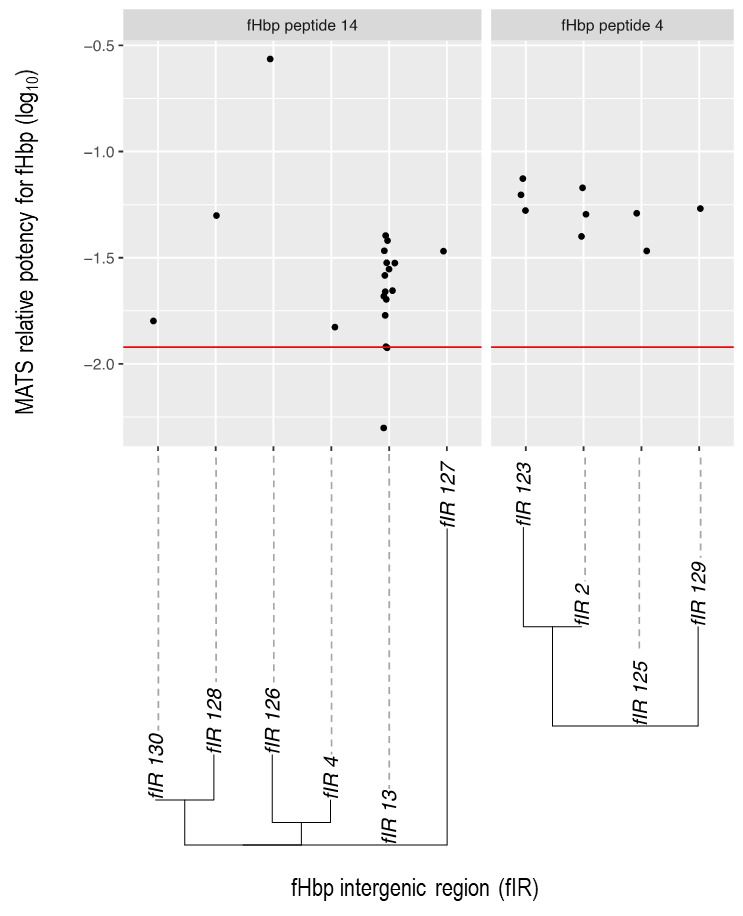

Supplement: FIG S5 [file mSphere.00376-20-sf005.docx]
