# Supplementary material for: Genomic Characterization of Invasive Meningococcal Serogroup B Isolates and Estimation of 4CMenB Vaccine Coverage in Finland
Source: mSphere. 2020 Sep 16;5(5):e00376-20. doi: 10.1128/mSphere.00376-20 (PMC7494829; doi:10.1128/mSphere.00376-20)
Supplement: TABLE S1 [file mSphere.00376-20-st001.docx]

|  | | MATS | |
| --- | --- | --- | --- |
|  |  | Covered | Not covered |
| gMATS | Covered | 45 | 3 |
|  | Unpredictable | 2 | 5 |
|  | Not covered | 0 | 5 |
