# Supplementary material for: Genomic Characterization of Invasive Meningococcal Serogroup B Isolates and Estimation of 4CMenB Vaccine Coverage in Finland
Source: mSphere. 2020 Sep 16;5(5):e00376-20. doi: 10.1128/mSphere.00376-20 (PMC7494829; doi:10.1128/mSphere.00376-20)
Supplement: Supplementary file 3 [file mSphere.00376-20-sf001.docx]

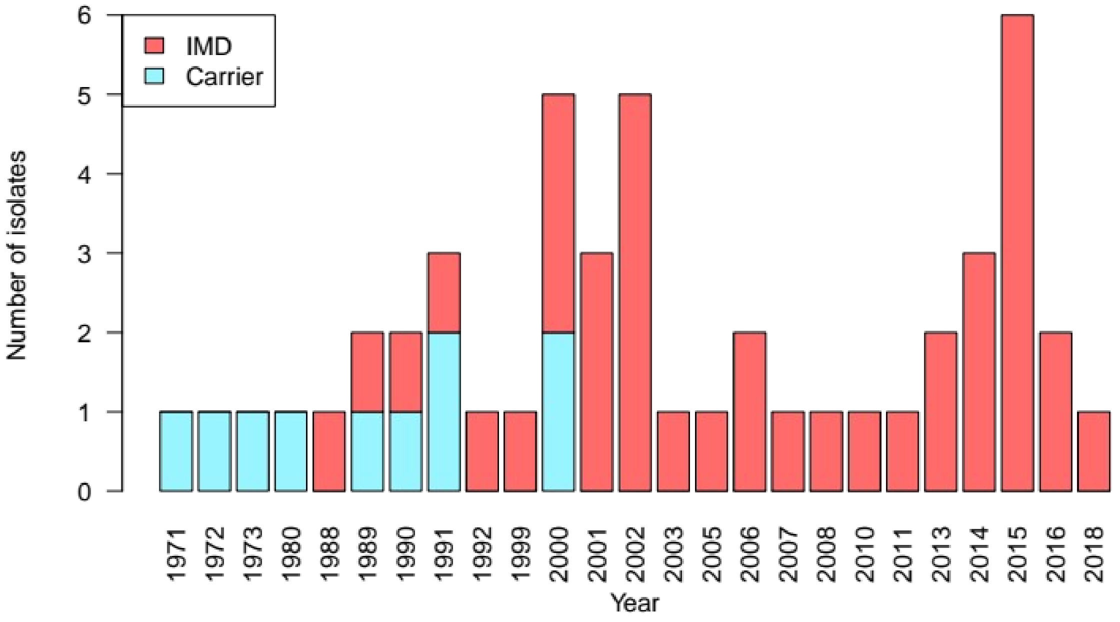
IMD
Carrier
Number of isolates
0
1
2
3
4
5
6
1971
1972
1973
1980
1988
1989
1990
1991
1992
1999
2000
2001
2002
2003
2005
2006
2007
2008
2010
2011
2013
2014
2015
2016
2018
Year
